# Effects of Music Intervention on Stress in Concussed and Non-Concussed Athletes †

**DOI:** 10.3390/brainsci11111501

**Published:** 2021-11-12

**Authors:** Camille Léonard, Jeanne Marie Desaulniers-Simon, Diana Tat, Louis De Beaumont, Nathalie Gosselin

**Affiliations:** 1 Department of Psychology, Université de Montréal, Montreal, QC H3T 1J4, Canada; camille.leonard@umontreal.ca (C.L.); diana.tat@umontreal.ca (D.T.); 2 International Laboratory for Brain, Music and Sound Research (BRAMS), Outremont, QC H2V 2S9, Canada; jeanne.marie.desaulniers-simon@umontreal.ca; 3 Music, Emotions and Cognition Research Laboratory (MUSEC), Université de Montréal, Montreal, QC H2V 2S9, Canada; 4 Faculty of Medicine, Université de Montréal, Montreal, QC H3T 1J4, Canada; 5 Department of Surgery, Université de Montréal, Montreal, QC H3T 1J4, Canada; louis.de.beaumont@umontreal.ca; 6 CIUSSS Nord-de-l’Ile-de Montréal Research Center, Hôpital du Sacré-Cœur-de-Montréal, Montreal, QC H4J 1C5, Canada; 7 Center for Research on Brain, Language and Music (CRBLM), McGill University, Montreal, QC H3G 2A8, Canada

**Keywords:** sport-related concussion, athletes, stress, musical intervention, music, emotions, mild traumatic brain injury

## Abstract

Sport-related concussion is a serious public health issue affecting millions of individuals each year. Among the many negative side effects, emotional symptoms, such as stress, are some of the most common. Stress management is repeatedly cited by expert groups as an important intervention for this population. It was shown that music has relaxing effects, reducing stress through the activation of brain areas involved in emotions and pleasure. The objective of this study was to explore the effects of a music-listening intervention compared with silence on experimentally induced stress in concussed and non-concussed athletes. To this aim, four groups of athletes (non-concussed music, non-concussed silence, concussed music, and concussed silence) performed the Trier Social Stress Test, for which both physiological (skin conductance level) and self-reported stress measurements were taken. No significant difference was found in the pattern of stress recovery for self-reported measurements. However, the skin conductance results showed greater and faster post-stress recovery after listening to music compared with silence for concussed athletes only. Taken together, these results suggest that music could be an efficient stress management tool to implement in the everyday life of concussed athletes to help them prevent stress accumulation.

## 1. Introduction

Concussion sustained in the context of competitive sports has been the subject of a growing body of literature, notably in regard to return-to-play guidelines as well as its long-term effects on health [1]. Concussion is a commonly used term in the scientific literature and in sports medicine to designate a mild traumatic brain injury that occurred during sporting activity [1,2]. Each year in the United States alone, between 1.6 million and 3.8 million concussions occur [3], a number that is underestimated according to several studies [4,5]. Estimates suggest that as many as 50% to 75% of concussions fail to be reported, on the grounds that athletes do not want to be suspended from the game or that the blow to the head is not serious enough to seek medical help [6,7].

A concussion is a complex pathophysiological process affecting the brain. It is induced by biomechanical forces, typically by a direct blow to the head, face, neck or elsewhere on the body with an impulsive force transmitted to the head, resulting in a loss of consciousness, amnesia, and/or neurological symptoms (e.g., confusion/disorientation) [1]. There are various physical (e.g., headache), cognitive (e.g., difficulty concentrating) and emotional (e.g., anxiety) symptoms that are associated with concussion [8,9]. Such clinical symptoms are generally resolved within an acute phase of 10 to 14 days following the trauma [1]. However, for 10–15% of athletes, symptoms persist beyond this acute phase [10], causing significant emotional distress, functional limitations, and delayed return to daily activities [11,12]. Previous research found that psychological reactions, such as stress and anxiety, were associated with developing and maintaining symptoms that persisted beyond the expected 10–14 day recovery period, suggesting a failure of normal clinical recovery [1,13,14,15].

Stress and anxiety are overlapping concepts that are commonly used in the concussion literature. Stress is a response of an organism when facing a situation with one of the following characteristics: novelty, unpredictability, threat to survival or ego, and/or low sense of control [16]. Anxiety is defined as an anticipation of future threat [17]. Although both reactions are interrelated, the anticipatory aspect of anxiety distinguishes it from stress, as anxiety is usually developed through repeated stress responses [18]. A stressful stimulus can induce both psychological (or emotional) and physiological responses, with the two influencing each other through interconnected neural networks [19]. The physiological response is mediated by the activation of the autonomic nervous system, which controls arousal and vital functions of the body. The autonomic nervous system includes the sympathetic nervous system (SNS); during a stress response, the SNS triggers the fight-or-flight response. The latter response provides the body with energy when faced with a stressor, causing, for example, elevation of heart rate, blood pressure, and sweating, allowing improved performance in cognitive and physical tasks [19,20]. Once the stressor has passed, stress recovery occurs; a decrease in arousal level allows the body to calm down and replenish the energy expended. Among physiological measurements, electrodermal activity is a commonly used measure in stress studies investigating the SNS. It represents changes in the electrical properties of the skin resulting from modulations in sweat secretion by the eccrine sweat glands, which are themselves innervated by the SNS. Indeed, the tonic component of the electrodermal conductance, also called skin conductance level (SCL), increases significantly in response to emotional changes induced by the perception of a stressful situation [21,22,23].

Concussions can generate various stressors for athletes: delayed return to sport, suboptimal sport performance, pressure from coaches, risk of sustaining another concussion, reduced quality of life or life satisfaction, reduced academic performance, fear of losing scholarship status, limited social support related to the invisible aspect of this type of injury, and more [15,24,25,26]. Concussion also commonly injures brain regions involved in emotion regulation (e.g., prefrontal cortex), which could alter stress regulation [13,27]. Moreover, several studies have shown that concussed athletes in the post-acute phase (from 14 days to several months after sustaining a concussion), whether symptomatic or asymptomatic, presented abnormalities in variations of SNS activity when exposed to a physical stressor, while reporting low subjective stress levels [28,29,30,31,32]. This indicates that athletes with a history of concussion, compared with their non-concussed peers, might present an altered physiological stress response, despite the resolution of clinical symptoms and low subjective stress levels. Thus, the literature indicates that concussed athletes are exposed to many stressors and that their stress response might be altered as a direct result of concussion in such a way that they are at risk of accumulating stress.

To this end, stress management is repeatedly cited as an important intervention for concussed athletes [1,12,15,24,33,34]. A relevant avenue to explore is the development of tools that help prevent the cumulative effects of stress and that are easy to implement in the everyday life of concussed athletes. The scientific literature and the sports community have a growing interest in the use of music, a strategy with demonstrated beneficial effects on stress and emotion regulation.

Music listening is already a part of athletes’ everyday lives; many athletes who believe music is important listen to it daily and report using it for the positive effect as well as to calm down [35]. This intuitive use of music is coherent with the fact that musical interventions are receiving a great deal of interest from researchers as evidenced by recent reviews and meta-analyses highlighting its beneficial effects on health [19,36,37]. The stress-reducing effects of music have been studied in both clinical and nonclinical populations [19,38]. In a laboratory setting, listening to music following stress induction was also shown to allow a faster stress recovery, compared with relaxation in silence [39,40,41,42,43] or listening to white noise [44].

Many studies have looked into the specific characteristics of music that may mediate its effects on stress. Low arousal music (i.e., relaxing music) is effective in reducing self-reported stress [38,45], heart rate [44,46], and electrodermal activity [47]. This is consistent with studies that showed that music listening activates brainstem structures which modulate autonomic responses [47,48]. A slow tempo (i.e., the speed of the beat) contributes to the relaxing effects by decreasing physiological reactivity [19]. Music with a tempo between 60 and 80 beats per minute was shown to be particularly effective in reducing stress [19]. Furthermore, music with positive emotional valence (i.e., pleasant music) also increases positive mood, which has stress-reducing effects [44,45]. A number of studies have shown that listening to pleasant music activates the limbic and paralimbic regions, which are related to emotional processing [19,49,50]. More specifically, music that induces very pleasant emotions can activate the meso-limbic circuit, a brain network associated with pleasure and reward [49,51,52]. Another element to consider is whether music chosen by researchers could be more effective in reducing stress than music chosen by participants since appropriate musical parameters for inducing a state of calm could be selected [38]. However, musical preference is not negligible in its stress reduction potential [45]. Indeed, an important factor in the induction of a state of relaxation is the subjects’ perception of the music as being relaxing [53].

In sum, the literature suggests that concussed athletes are exposed to numerous stressors and that their stress response might be altered as a direct result of concussion. Hence, they are at risk of accumulating stress despite the resolution of clinical symptoms and low subjective stress levels. Considering that stress management is emphasized in numerous concussion guidelines and that music listening has been shown to be beneficial in various populations, such an intervention could help concussed athletes unwind after being exposed to a stressor, potentially preventing stress accumulation. Music interventions are non-invasive, cost effective, and simple to use for athletes [19,54,55] who consider music to be important in their everyday life.

Therefore, the main goal of this study was to examine the effects of musical intervention on physiological (i.e., skin conductance level) and self-reported stress measurements in concussed and non-concussed athletes following a stressor. More specifically, the objective was to determine the effects of listening to relaxing and pleasant music in comparison to resting without intervention (in silence) on stress measurements during a defined post-stress period, using a laboratory-induced stress protocol. It was hypothesized that the music intervention would allow greater reduction in skin conductance level and self-reported stress measurements than resting in silence for all athletes. Moreover, concussed athletes might be more sensitive to induced stress than their non-concussed peers, and might therefore show a greater potential to benefit from musical intervention.

## 2. Materials and Methods

### 2.1. Participants

In total, 84 participants between 18 and 35 years of age matched our eligibility criteria. To participate, they had to be considered elite and highly competitive athletes; the pursuit of excellence and high-level performance in their sport had to be a significant part of their daily lives. On average, athletes trained 9.66 h per week (*SD* = 5.83) and had 18 competitions per year (*SD* = 17.41). Exclusion criteria for the study consisted of diagnosed hearing impairment, neurological disorders (e.g., epilepsy), or psychiatric disorders (e.g., current anxiety or depression episode), evaluated in a clinical interview with participants. Five participants with attention deficit disorder were included. At the time of their testing, no participant was taking psychotropic drugs. In the present nonrandomized controlled trial, concussed (*n* = 33) and non-concussed (*n* = 51) athletes were alternately assigned to a musical intervention (music groups) and to a no-intervention condition (silence groups).

All participants filled out French adaptations of affective questionnaires to compare groups on pre-existing affective symptoms since it is known that anxiety can influence reactivity to stress [56]. First, the Perceived Stress Scale-10 items was used, which measures global perception of stress (the degree to which situations in one’s life are appraised as stressful) [57,58]. The level of state anxiety (i.e., anxiety at a specific moment) and trait anxiety (i.e., predisposition to perceive the environment as more stressful) were documented through the State-Trait Anxiety Inventory [59,60]. Higher scores for each of the questionnaires indicated higher levels of stress and anxiety, respectively.

For concussed athletes, detailed information regarding the number of previous concussions, their approximate date, the description of each accident, and the severity markers (loss of consciousness, amnesia, confusion/disorientation) were obtained through a validated, semi-structured clinical interview [61]. Their last concussion had to have occurred at least three months prior to participation. Concussed participants filled out the Rivermead Post-Concussion Symptoms Questionnaire to assess the intensity of specific symptoms (on a 4-point scale) associated with concussion felt over the last 24 h [62]. At the time of the study, no participant showed significant post-concussion symptoms, according to the Rivermead clinical threshold (over 16/64) [63].

Table 1 presents the characteristics of participants stratified by group. All groups were equivalent in terms of demographic characteristics (sex, age, years of education, and years of music training) and affective symptoms (stress, state anxiety, trait anxiety). The concussed groups were equivalent in terms of delay since latest concussion and persistent post-concussive symptoms but tended to be different in terms of the number of previous concussions. No covariance was found between this variable and skin conductance level or self-reported stress; thus, it was not included in the subsequent analyses.

This study was approved by the Arts and Sciences Research Ethics Committee of Université de Montréal, and by the Committee of Ethics in Research of Collège Montmorency.

### 2.2. Study Procedure

**Figure 1:**
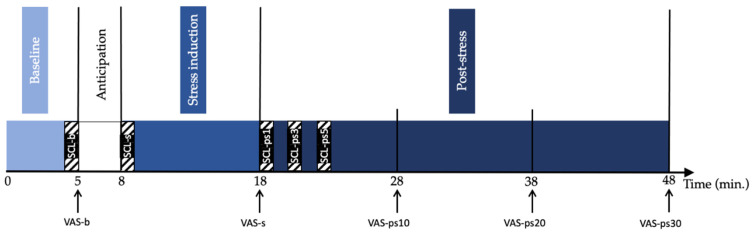
Trier Social Stress Test procedure. Hatched rectangles represent one-minute epochs where skin conductance level was extracted, and arrows represent moments where self-reported stress measurements were taken. SCL-b = SCL at baseline. SCL-s = SCL at stress induction. SCL-ps1, SCL-ps3 and SCL-ps5 = SCL at 1, 3 and 5 min post-stress, respectively. VAS-b = self-reported stress at baseline. VAS-s = self-reported stress after stress induction. VAS-ps10, VAS-ps20 and VAS-ps30 = self-reported stress at 10, 20 and 30 min post-stress, respectively. SCL = skin conductance level. VAS = visual analogue scale.

Upon arrival at the laboratory and after providing written consent, participants were asked to sit alone in the testing room for 30 min while filling out demographic and affective questionnaires. Then, electrodes were placed to record their skin conductance level (SCL) throughout the experiment. Stress was induced using the Trier Social Stress Test (TSST) procedure [64]. The TSST procedure took place in the following manner (see Figure 1):

Baseline. The participants were instructed to remain calmly seated for five minutes. After this delay, they were asked to evaluate their stress level (VAS-b).

Anticipation. The participants had three minutes to mentally prepare a speech explaining why they would be the perfect candidate for their chosen job. They were also told that the interview would be filmed.

Stress induction. The participants performed a five-minute speech in front of the experimenter and a camcorder. The experimenter took notes while looking at the participant with a neutral facial expression. After the speech, they completed a mental arithmetic test for a period of five minutes. After stress induction, the participants were asked to rate their stress level (VAS-s).

Post-stress recovery. The participants listened to relaxing and pleasant music or remained in silence (depending on the assigned group) using Beyerdynamics DT990PRO headphones while relaxing with their eyes closed. The participants in the silence groups were also asked to wear headphones. Every 10 min during the recovery phase, the participants were asked to evaluate their stress level (VAS-ps10, VAS-ps20 and VASps-30).

Finally, after the TSST procedure, the participants rated the arousal and emotional valence of each musical excerpt they had listened to (see Section 2.4).

### 2.3. Measures

#### 2.3.1. Self-Reported Stress Measurements

Self-reported stress was measured with visual analogue scales (VAS) shown on the computer screen. The VAS is a 100 mm straight horizontal line whose ends are defined as the extreme limits of the parameter to be measured. The participants were asked to click on the line at the point of the VAS that best corresponded to the intensity of the stress being felt at the time, from “No stress’’ (0) to ‘’Severe stress’’ (100). Self-reported stress measurements were taken five times during the TSST protocol: at the end of baseline, at the end of stress induction, and at 10, 20 and 30 min post stress (see Figure 1).

#### 2.3.2. Skin Conductance Level

Electrodermal conductance was recorded continuously during the TSST using two F-E5SH-62 surface electrodes (Natus Neurology Incorporated, Pleasanton, CA, U.S.A.) coated with Gel 101 (Isotonic Recording Electrode Gel, Biopac Systems Inc., Goleta, CA, U.S.A.) and connected to the GSR 100C module of the Biopac MP150 system. The index and middle fingers of the participant’s non-dominant hand were cleaned with distilled water, and electrodes were placed on the palmar surface of the proximal interphalangeal joints of those fingers. AcqKnowledge software (version 5.0, Biopac Systems Inc., Goleta, CA, U.S.A.) was used for the analysis of tonic levels of electrodermal activity, also known as the skin conductance level. A 0.05 Hz bandpass was used to isolate the SCL. SCL was then extracted in one-minute windows during the TSST phases of interest: at baseline (last minute), during stress induction (first minute) and during recovery (first, third and fifth minutes) as shown in Figure 1. For each measurement time, a participant’s SCL was transformed into a standard *z* score, using the average of all electrodermal conductance data from that same participant, in order to facilitate inter-subject comparison [22,65]. The *z* scores of all subjects in the same group were then averaged for the 5 phases of interest of the TSST.

### 2.4. Musical Material

For the music groups, six instrumental music excerpts were selected from the classical repertoire through interrater agreement between three experimenters (e.g., Clarinet Concerto in A, II. Adagio, by Mozart) [66]. They all agreed on the following characteristics: (1) low arousal (relaxing); (2) positive emotional valence (pleasant); (3) low tempo (*M* = 60, *SD* = 12); and (4) major mode.

Music excerpts were evaluated in terms of arousal and valence by all our music group participants (NCM and CM) at the end of the TSST. Using a mouse, the participants clicked on the area of the visual analogue scale that corresponded to their level of arousal (0 = very relaxing, 100 = very stimulating) and valence (0 = very unpleasant, 100 = very pleasant) for each musical excerpt. Both groups judged the musical stimuli as relaxing and pleasant; however, athletes in the CM group found the musical excerpts more pleasant and relaxing than those in the NCM group (see Table 2).

### 2.5. Data Analysis

For self-reported stress measurements, a 2 population (concussed, non-concussed) X 2 condition (music, silence) X 5 time points (baseline, stress induction, 5 min post-stress, 10 min post-stress, and 30 min post-stress) mixed ANOVA was performed, using VAS measurements (possible values between 0 and 100).

Similarly, for skin conductance level, a 2 population (concussed, non-concussed) X 2 condition (music, silence) X 5 time points (baseline, stress induction, 1 min post-stress, 3 min post-stress, and 5 min post-stress) mixed ANOVA was conducted, using standardized SCL values (*z* scores). Then, 5 two-way ANOVAs were executed to assess simple two-way interactions between condition and population (that is, differences between groups at the five measurement times independently). Finally, 4 one-way repeated measures ANOVAs were completed to assess differences within groups over time.

For SCL and self-reported stress measurements, all pairwise comparisons were performed for statistically significant simple main effects. This allowed the effectiveness of stress induction and the effect of music to be determined, as well as comparing the four groups in terms of self-reported measurements and skin conductance level at different time points during the TSST procedure. For all analyses, Greenhouse–Geisser correction was used when the assumption of sphericity was violated. Bonferroni corrections were applied for each two-way ANOVA and simple main effect.

As all correlations between (a) the number of concussions, and (b) self-reported stress measurements and skin conductance levels were not significant (*p* > 0.05), those analyses are not presented in the following Results.

## 3. Results

### 3.1. Self-Reported Stress Levels (VAS)

**Figure 2:**
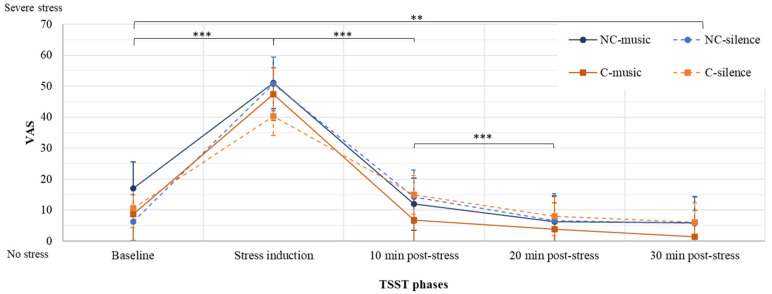
VAS measurements at each time point during the TSST protocol, presented as a function of group (mean ± standard error of the mean). *** *p* ≤ 0.001. ** *p* = 0.006.

As shown in Figure 2, a three-way mixed ANOVA showed no statistically significant interaction between population, condition and time on the VAS (*F*(3, 214) = 1.58, *p* = 0.200, partial η^2^ = 0.019). Additionally, no statistically significant two-way interactions were observed between population and time (*p* = 0.499), population and condition (*p* = 0.381), or condition and time (*p* = 0.087). However, there was a statistically significant simple main effect of time on the VAS (*F*(3, 214) = 161.720, *p* < 0.001, partial η^2^ = 0.669). No other simple main effects were noted (*p* > 0.05).

Subsequent pairwise comparisons showed that self-reported stress increased significantly between baseline and stress induction for all participants (*p* < 0.001)*,* indicating that stress was successfully induced. There was also a significant decrease in VAS measurements between stress induction and ten minutes post-stress *(p* < 0.001)*,* a trend that was maintained between 10 min and 20 min post-stress (*p* < 0.001). Then, stress levels were stable between 20 min and 30 min post-stress *(p* = 1.00). Moreover, compared with baseline, participants had significantly lower stress levels at the end of the recovery phase (*p* = 0.006).

### 3.2. Skin Conductance Level (SCL)

**Figure 3:**
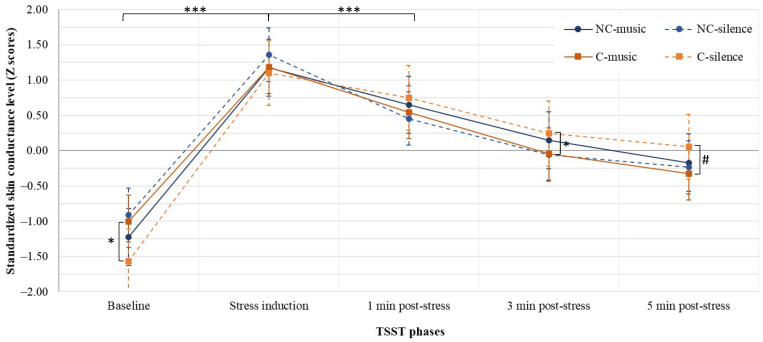
Standardized SCL extracted at each time point during the TSST procedure, presented as a function of group (mean ± standard error of the mean). *** *p* ≤ 0.001. * *p* ≤ 0.05. # *p* = 0.059.

The skin conductance level for each group is presented in Figure 3. A three-way mixed ANOVA showed a significant three-way interaction between time, population, and condition for the skin conductance level (*F*(3,203) = 4.627, *p* = 0.006, partial η^2^ = 0.055), as well as a significant main effect of time (*F*(3,203) = 162.665, *p* < 0.001, partial η^2^ = 0.670).

#### 3.2.1. Differences between Groups

A two-way ANOVA showed a significant interaction between Population and Condition on SCL at baseline (*F*(1,80) = 6.553, *p* = 0.012, partial η^2^ = 3.849) and at the third minute post-stress (*F*(1,80) = 8.024, *p* = 0.006, partial η^2^ = 1.226). At baseline, SCL was significantly lower in athletes in the CS group, compared with the CM group (*M* = −1.568, *SD* = 0.192 and *M* = −1.001, *SD* = 0.186, respectively), *p* = 0.037. However, there was a significant effect of music on concussed athletes at three minutes post-stress; the mean standardized SCL was lower in the CM group (*M* = −0.047, *SD* = 0.095) than in the CS group (*M* = 0.243, *SD* = 0.098), *p* = 0.036. A tendency in ANOVA and a simple main effect were found at the fifth minute post-stress (*F*(1,80) = 3.684, *p* = 0.059, partial η^2^ = 1.009); the mean standardized SCL was lower in the CM group (*M* = −0.326, *SD* = 0.127) than in the CS group (*M* = 0.055, *SD* = 0.131), *p* = 0.040. There were no significant two-way interactions at other TSST phases: stress induction (*F*(1,80) = 0.798, *p* = 0.374, partial η^2^ = 0.359) or one minute post-stress (*F*(1,80) = 3.499, *p* = 0.065, partial η^2^ = 0.812). Note that no significant two-way interactions or simple main effects were identified at any measurement time for non-concussed groups.

#### 3.2.2. Differences within Groups

One-way repeated measures ANOVAs showed statistically significant variations in SCL during the TSST for all groups: NCM, *F*(2,1) = 63.241, *p* < 0.001, partial η^2^ = 0.709; NCS, *F*(3,59) = 39.272, *p* < 0.001, partial η^2^ = 0.631; CM, *F*(2,1) = 63.241, *p* < 0.001, partial η^2^ = 0.709; CS, *F*(2,28) = 65.005, *p* < 0.001, partial η^2^ = 0.913. First, the post hoc comparisons showed a significant increase in SCL between baseline and stress induction for each group (all *p*-values < 0.001).

For both concussed groups (CM and CS), no significant decrease in their physiological stress level was noted between stress induction and the first minute post-stress (*p* = 0.075 and *p* = 1.000, respectively). The SCL then decreased significantly between the first and the third minutes post-stress (CM, *p* = 0.003; CS, *p* < 0.001), but not between the third and the fifth minutes post-stress (CM, *p* = 0.077; CS, *p* = 1.000). The SCL at the third and fifth minutes post-stress did not significantly differ from the baseline for CM athletes (*p* > 0.058), but did for CS athletes (*p* < 0.001). CS athletes showed a significantly higher physiological stress level at the third and fifth minutes, when compared to their baseline.

The SCL of non-concussed athletes in the silence group (NCS) was significantly lower at the first minute post-stress, compared to the moment of stress induction (*p* = 0.001), which was not the case for athletes in the music group (NCM, *p* = 0.055). The two groups had a statistically significant decrease in SCL from the first minute post-stress to the third minute post-stress (*p* < 0.001). Only the NCM group showed a significant difference between the third minute post-stress and the fifth minute post-stress (*p* < 0.001). The SCL at these two collection points was statistically higher from the baseline SCL for NCM athletes (*p* < 0.001). This was only the case at the third minute post-stress for NCS athletes (*p* = 0.018).

## 4. Discussion

The aim of this study was to examine the effects of a music intervention following a laboratory-induced stressor on self-reported stress and skin conductance level in concussed and non-concussed athletes. We hypothesized that athletes who listened to music would show lower stress levels during the post-stress period when compared to a silence condition. Moreover, concussed athletes could potentially benefit more from musical intervention, as they might be more sensitive to stress during the protocol than their non-concussed peers.

In brief, results confirmed that stress was successfully induced in participants using the TSST; all groups had a significant increase in the two measurements (VAS and SCL) between baseline and the stress induction phase. Each group then exhibited a decrease in their self-reported and psychophysiological stress levels over time, as expected [67,68,69]. Globally, a difference was noted in patterns of post-stress recovery between experimental groups for skin conductance level, whereas this was not the case for self-reported stress.

Firstly, although concussed athletes did not seem to have reached higher stress levels during the TSST, comparisons between SCL of concussed and non-concussed athletes in the silence groups demonstrated that post-stress recovery was slower among concussed athletes than their non-concussed peers. In fact, SCL started to decrease at the end of stress induction for the NCS group, whereas the same phenomenon was delayed in the post-stress phase for the CS group, suggesting less effective stress management capacities for this clinical population. These results are consistent with the literature indicating that stress regulation difficulties can persist, even if the athletes are no longer in the acute post-concussion phase [28,29,30,31,32]. On the self-reported stress measure, however, concussed athletes did not report being more stressed than non-concussed counterparts at any moment of the TSST procedure. Taken together, these results are congruent with previous findings that athletes with a history of concussion appear to present altered physiological stress responses when faced with stressors, despite low self-reported stress levels [29]. It should be noted that it is possible for self-reported stress levels to be discordant with the physiological stress levels of athletes, as they are a representation of their perception of the stress experienced. Moreover, such discordance was found in other studies utilizing laboratory-induced stress [70,71,72].

Secondly, differences between recovery patterns in skin conductance level for the two groups of concussed participants were noted. Even though they both had a similar mean skin conductance level at the first minute of recovery, concussed athletes who listened to music then showed a greater decrease in their physiological stress than those in the silent group, attested by lower SCL values for the CM group than for the CS group at the third minute post-stress. This difference tended to be maintained at the fifth minute post-stress. Concordantly, only the CM group returned to its baseline level, suggesting a full post-stress recovery. These results indicated that recovering from exposure to a stressor while listening to music was more effective in reducing skin conductance level than recovering in silence. Such results confirm the findings in the literature regarding the effects of music on stress when compared with the absence of intervention [39,40,41,42,43,73,74].

In comparison, no significant difference in standardized skin conductance level was found between non-concussed groups. Both conditions showed a significant SCL decrease to baseline levels during the first five minutes post-stress, indicating that the non-concussed athletes showed similar stress levels during the post-stress period whether they listened to music or remained in silence. Healthy athletes are known to be less reactive to induced stress than non-athletes, as demonstrated by an attenuated physiological response following the TSST [69,75]. It was also proposed that athletes recover more effectively from stress, compared with the general population, possibly from the repeated exposure to stress during the practice of their sport discipline [69]. Those stress regulation capacities could play a more important role than the relaxing properties of music and could explain, in part, the lack of significant difference between the two groups.

Thus, the differences found between concussed and non-concussed athletes may be explained by the fact that healthy athletes have excellent capacities for dealing with stressors, while the stress response of concussed athletes might be altered as a direct result of the concussion. Consequently, it may be that for non-concussed athletes, musical intervention is not necessary to obtain an effective recovery, whereas its benefits are noticeable for the concussed population with poorer stress management.

Furthermore, although all participants in the music listening groups rated the musical excerpts in terms of emotional valence and arousal, athletes in the CM group found the excerpts more pleasant and relaxing than those in the NCM group. These different emotional judgments of the musical material might explain in part the different stress recovery pattern found between the CM and NCM groups. Musical preference may also have an impact on the relaxing potential of music [45]. It is possible that classical music was not the style preferred by the athletes in this study, who usually listen to music to increase their motivation and their performance (more activating than relaxing) [37,76], thus decreasing its impact on stress. Indeed, research has shown that most athletes prefer rock (32%), hip-hop (28%) and pop (25%), while classical music was chosen by only 1% of the sample [35]. In addition, athletes usually prefer music with a moderate to fast tempo [77,78], whereas the music presented in this study was composed with a slow tempo. This potential difference between the music presented and the personal preferences of the athletes surveyed could have decreased the impact of the presented music on stress.

The groups of concussed athletes tended to be heterogeneous in the number of concussions per athlete. This difference could partly explain the variability in the athletes’ stress regulation during the TSST protocol. While the cumulative effect of multiple concussions could have long-term neurological impact [5,79], whether there is any type of cumulative effect on the stress management capacities of athletes is not clear. Indeed, the stress levels of the CM group during stress induction were not higher than that of the average CS group participant, but music intervention seemed to have a greater effect on stress levels for participants in the CM group, who tended to have a higher average number of concussions. Further research is necessary to ascertain the impact of this variable on stress regulation.

This study has several limitations. The small sample size limited the interpretation of the results; hence, further research with a larger sample is needed to validate these preliminary findings. Additionally, greater control over the selection of participants would be preferable, as differences between and within groups (such as number of concussions) may influence individual responses to stress and music. The opportunity to account for the type of sport played and the competitive level would be beneficial. Even if our groups are comparable in terms of time period since the last concussion, it would be interesting to see if similar results can be achieved with a shorter and more homogeneous timeframe.

Among the strengths of this study, the use of both psychophysiological and self-report measurements is worth mentioning; the utilization of self-report measures alone would have given us a largely incomplete picture. Furthermore, the controlled design enabled the comparison of musical intervention effects in both clinical and non-clinical populations. The inclusion of tools allowing the comparison of affective symptoms between groups was also a strength of our protocol since it ensured that the groups were similar in terms of pre-existing stress and anxiety. Moreover, this study compliments the existing literature on the effects of music on stress in terms of study environment for clinical populations, which were mostly explored in medical settings.

For further exploration, an acoustic control condition that is not music should be included. Adding a comparison group that recovers from stress while resting with another type of acoustic stimulation (e.g., audio book) would strengthen the interpretations of the possible beneficial effects found for music listening. Moreover, given that music preference plays a key role in music appreciation, an interesting alternative to consider in the future would be to have the participants select the music. Additionally, the use of varied measures of stress (e.g., heart rate, cortisol) would be pertinent to document the effects of music listening on different markers of human stress, allowing us to draw even more precise conclusions. It would also be interesting to explore the effects of music listening prior to a stressor in athletes, as studies have shown that it can lower stress reactivity [73,74]. Each of these proposed future directions would contribute to the documentation of stress in concussed and non-concussed athletes, as well as the development of an effective intervention that could be used every day to promote stress recovery.

## 5. Conclusions

In conclusion, the findings of this study indicate that listening to music is effective in reducing the physiological response of concussed athletes following the presentation of a stressor. Despite the limitations of this study, the results suggest that music interventions could possibly help this clinical population during stress recovery, in line with the literature supporting this approach in other populations. Further research is necessary to confirm these findings, but this study provides a good starting point for discussion and a good basis for further research that could eventually contribute to the development of new tools to help concussed athletes recover optimally from stress, as they are particularly at risk of accumulating stress following concussions.

## Figures and Tables

**Table 1:** Characteristics of participants in each group.

	Variables	NCM (*n* = 27)	NCS (*n* = 24)	CM (*n* = 17)	CS (*n* = 16)	*p*-Value
Demographic characteristics	Sex (women/men)	14/13	9/15	7/10	7/9	0.767
	Age (years)	20.89 (1.48)	22.00 (2.96)	22.76 (3.47)	23.81 (4.98)	0.235
	Years of education	16.83 (1.93)	16.92 (2.50)	16.65 (2.18)	16.27 (2.52)	0.833
	Years of music training	2.30 (3.01)	1.67 (2.88)	1.43 (2.36)	2.13 (3.20)	0.750
Sport practice	Type of sports (contact/team with reduced contact/aquatic/endurance/ ball-racket/balance-coordination)	4/4/10/4/4/1	9/0/2/8/5/0	11/2/1/1/0/2	5/2/1/5/0/3	-
Affective symptoms	PSS-10 (stress, max score = 40)	13.22 (5.98)	14.04 (4.89)	14.06 (5.36)	14.50 (6.61)	0.899
	STAI-State (anxiety, max score = 80)	32.07 (7.65)	33.04 (7.49)	33.24 (6.05)	30.50 (5.03)	0.634
	STAI-Trait (anxiety, max score = 80)	37.67 (6.93)	37.92 (7.17)	36.35 (6.05)	35.63 (5.48)	0.663
Concussion information	Number of previous concussions	-	-	2.88 (1.69)	1.88 (1.20)	0.059
	Delay since latest concussion (months)	-	-	33.80 (20.48)	51.53 (54.08)	0.250
	Rivermead (persistent post-concussive symptoms)	-	-	6.47 (4.97)	6.93 (5.80)	0.815

Numbers shown above are mean (standard deviation), except for sex and type of sports, for which frequency is shown. *p*-values were obtained with one-way ANOVA, except for sex, where *p*-values were obtained with Chi-square test. NCM = non-concussed music. NCS = non-concussed silence. CM = concussed music. CS = concussed silence. PSS-10 = Perceived Stress Scale. STAI-State/Trait = State-Trait Anxiety Inventory.

**Table 2:** Judgments of the valence and arousal of music excerpts in the non-concussed music (NCM) and concussed music (CM) groups.

	NCM	CM	*p*-Value
Valence	71.89 (16.05)	84.27 (14.91)	0.019 *
Arousal	22.00 (10.40)	13.73 (12.29)	0.026 *

Numbers shown above are means (standard deviations) and compared using independent *t*-tests. * *p* < 0.05. Valence judgments ranged from 0 = very unpleasant to 100 = very pleasant and arousal judgments ranged from 0 = very relaxing to 100 = very stimulating.

## Data Availability

Study participant were assured that raw data would remain confidential and would not be shared.
